# Metabolic Engineering of *Bacillus licheniformis* for High-Yield L-Lactic Acid and Galactooligosaccharide Retention in Complementary Synbiotics Production

**DOI:** 10.3390/microorganisms13112530

**Published:** 2025-11-04

**Authors:** Jihua Zhao, Teng Mu, Dandan Niu, Zhongzhen Ding, Nokuthula Peace Mchunu, Meng Zhang, Suren Singh, Zhengxiang Wang

**Affiliations:** 1 Research Center for Green Biomanufacturing, College of Chemical Engineering and Materials Science, Tianjin University of Science and Technology, Tianjin 300457, China; zhaojihua26@163.com (J.Z.); mt15832315529@163.com (T.M.); np.mchunu@nrf.ac.za (N.P.M.); 98213643@tust.edu.cn (M.Z.); 2 Tianjin Key Laboratory of Industrial Microbiology, College of Biotechnology, Tianjin University of Science and Technology, Tianjin 300457, China; 17860260614@163.com; 3 School of Life Science, University of KwaZulu Natal, Durban 4000, South Africa; 4 Department of Biotechnology and Food Science, Faculty of Applied Sciences, Durban University of Technology, P. O. Box 1334, Durban 4001, South Africa; singhs@dut.ac.za

**Keywords:** metabolic engineering, *Bacillus licheniformis*, complementary synbiotics, galactooligosaccharides, L-lactic acid

## Abstract

Using *Bacillus licheniformis* H107 as the initial strain, a novel complementary synbiotics production method was developed through comprehensive metabolic engineering strategies. Key modifications included the systematic analysis and reconstruction of the central carbon metabolism pathway through precise gene editing, targeting the deletion of *pflB, alsS, ydaP,* and *pycA* genes while disrupting *ganA1* and *ganA2* to block galactooligosaccharide (GOS) metabolism. Additionally, heterologous expression of the L-lactate dehydrogenase gene (*BcoaLDH*) was implemented, resulting in the engineered strain H107-06A. Shake-flask fermentation under anaerobic conditions with 20 g/L glucose yielded L-lactic acid production of 4.45 g/L, representing a 15.3-fold increase compared to the wild type. In a 5 L fermenter using GOS syrup as the carbon source, the engineered system synergistically produced complementary synbiotics, comprising L-lactic acid (42.56 g/L), GOS (141.89 g/L, accounting for 75.09% of total sugars), and viable cells (3.82 g/L). These findings provide a foundation for developing innovative and functional fermentation products.

## 1. Introduction

The synergistic application of probiotics and prebiotics, collectively termed synbiotics, has garnered increasing attention for their potential to improve gut health and overall well-being [1,2,3]. Synbiotics stand out due to their unique ability to combine live microbial strains with functional substrates, conferring independent but complementary physiological activities [4,5]. Among prebiotics, galactooligosaccharides (GOS) exemplify functional efficacy thanks to their stability and exceptional physiochemical properties, enabling the selective promotion of beneficial gut bacteria [5,6,7]. However, current strategies for synbiotic production often rely heavily on the simple co-blending of probiotics and GOS, which lacks innovative integration or industrial scalability [2,8].

A novel scientific challenge emerges to overcome these limitations with biotechnologically advanced approaches capable of synergistically producing probiotics, GOS, and functional by-products through microbiological and metabolic engineering.

Commercially, GOS is traditionally produced through the enzymatic transglycosylation of lactose using β-galactosidase, during which glucose is unavoidably generated as a by-product [9]. This raises a pivotal question: can metabolic engineering be employed to construct specialized microbial strains capable of converting this glucose into valuable functional products, thereby developing a new generation of complementary synbiotics with enhanced functionality and economic feasibility?

*Bacillus licheniformis*, a widely recognized industrial microorganism, is characterized by high metabolic activity and a robust safety profile [10,11,12]. This species efficiently metabolizes simple sugars like glucose and offers the potential for producing functional products through metabolic engineering [13,14]. Among these, L-lactic acid stands out for its ability to lower intestinal pH and inhibit harmful bacterial growth [15,16,17]. Thus, constructing a strain with selective glucose metabolism and efficient L-lactic acid production could enable the combined production of high-purity GOS, viable cells, and functional metabolites. Nonetheless, the native *B. licheniformis* strain faces two major challenges: its intrinsic ability to degrade GOS, which prevents GOS retention, and its limited capacity for L-lactic acid synthesis due to the complex nature of pyruvate metabolism [18,19]. Genomic studies suggest that these challenges stem from β-galactosidase gene expression and multiple pyruvate metabolic branches. While single-gene manipulations have shown incremental improvements [20], the coordinated optimization of these pathways for efficient GOS retention and L-lactic acid production remains unexplored.

In this study, using metabolic engineering strategies, we systematically deleted the β-galactosidase genes and pyruvate metabolism branch genes in *B. licheniformis* H107 while introducing the exogenous L-lactate dehydrogenase gene, resulting in an engineered strain with selective metabolic and efficient L-lactic acid synthesis capabilities. This strain can selectively utilize the glucose in GOS syrup, converting it into L-lactic acid and viable cells while retaining GOS, achieving the synergistic production of three functional components. This process not only provides a novel preparation method for complementary synbiotics but also offers new ideas for innovative development in the field of fermentation engineering.

## 2. Materials and Methods

### 2.1. Strains, Vectors, Primers, and Chemicals

The strains and plasmids used in this study are listed in Table 1. *Escherichia coli* JM109 was used as the host cell for DNA manipulation and plasmid DNA preparation. *B. licheniformis* H107 was used as the wild type of the mutant strain in this study. Plasmid pUB-EX was a thermosensitive *E. coli*-*Bacillus* shuttle vector [21,22], which was utilized for markerless gene deletion in *B. licheniformis*. The plasmid pHY-P43-BcoaLDH cloned the encoding gene *BcoaLDH* of L-lactate dehydrogenase in *Bacillus coagulans*, which can enhance the synthesis and secretion of L-lactic acid in cells [23]. Cultivation of the strains was conducted at 37 °C and 220 rpm for 12–16 h in Luria–Bertani (LB) broth or LB plates at 37 °C. The media were supplemented with 100 µg/mL ampicillin or 20 µg/mL kanamycin when required. All oligonucleotide primers for gene amplification were synthesized by Shenggong Biotech (Shanghai) Co., Ltd., Shanghai, China; details are provided in Table S1. Galactooligosaccharide syrup was prepared in our laboratory with a concentration of 600 g/L, among which the glucose concentration was about 130 g/L [9].

### 2.2. Gene Deletion and Construction of Recombinant Bacteria

Plasmid DNA extraction, PCR amplification of DNA fragments, DNA recovery, restriction digestion, ligation, and identification of transformants were carried out according to standard genetic manipulation techniques [24]. Genetic transformation of *B. licheniformis* H107 was performed by electrotransformation as previously described [25]. The deletion of specific genes in *B. licheniformis* (*pflB*, *alsS*, *ydaP*, *pycA*, *ganA1*, and *ganA2*) was performed using homologous recombination techniques as described in prior studies [22]. Gene deletion experiments utilized upstream and downstream homologous arms of the target gene, amplified by PCR using genomic DNA from *B. licheniformis* H107. Deletion fragments, constructed via overlapping PCR, were cloned into the *Sma* I and *Xba* I sites of plasmid pUB-EX [21,22]. Recombinant plasmids were introduced into *B. licheniformis* through electroporation. Homologous recombination events were induced by cultivating transformants at elevated temperatures (42–45 °C), followed by screening for single- and double-crossover events to isolate mutant strains [21,22].

### 2.3. Flask Fermentation Experiments

The flask fermentation experiments were carried out according to the previous publications [26,27] in 250 mL flasks with the working volume of 50 mL. Briefly, cells were grown in 50 mL of LB medium in a 250 mL flask at 37 °C with shaking (200 rpm) for 10–12 h until a cell density (OD_600_) of 2.5–3.0 was reached. Cells were collected by centrifugation and resuspended in a modified M9 medium and then inoculated into 50 mL of the modified M9 medium complemented with 5 g/L glucose in a 250 mL flask with the initial cell density (OD_600_) of 0.15. For cell growth experiments, the cultivation was carried out at shaking speed of 200 rpm and at 37 °C. For lactate fermentation, the cultivation was first carried out at 37 °C and 200 rpm for 12 h, then 20 g/L glucose was added, followed by stationary cultivation (anaerobic fermentation) for lactate formation at 37 °C. Calcium carbonate with a final concentration of 40 g/L was added for neutralization. Sampling was carried out during the cultivation. Modified M9 medium contained (per liter) 15.11 g Na_2_HPO_4_·12H_2_O, 3 g KH_2_PO_4_, 1 g NH_4_Cl, and 0.5 g NaCl. A total of 1 mL of filter-sterilized 1 M MgSO_4_ and 1 mL of filter-sterilized trace element solution containing (per liter) 2.4 g FeCl_3_·6H_2_O, 0.3 g CoCl_2_·6H_2_O, 0.15 g CuCl_2_·2H_2_O, 0.3 g ZnCl_2_, 0.3g Na_2_MO_4_·2H_2_O, 0.075 g H_3_BO_3_, and 0.495 g MnCl_2_·4H_2_O was added to a liter of the final medium [26,27].

### 2.4. Bioreactor Experiments

A fed-batch fermentation experiment in a bioreactor was carried out according to the method described [27,28]. A 5 L bioreactor (Biotech-5bg; Baoxing Saisi Pharmaceutical Equipment (Shanghai) Co., Ltd., Shanghai, China), initially containing 2.2 L of the modified M9 medium as described above in the “Flask Fermentation Experiments” Section, was used for L-lactate production from GOS syrup. The two-phase fed-batch process was started by inoculating 200 mL fresh inoculum prepared by preculturing cells in fresh modified M9 medium as described above. The cells were cultivated in aerobic conditions followed by anaerobic fermentation. During the aerobic phase, GOS syrup was supplemented with 24 g/L for cell growth. The culture was grown at pH 6.5, controlled by automatically feeding 25% (*w*/*v*) NH_4_OH solution, and the dissolved oxygen tension was maintained at above 30% of saturation. Anaerobic fermentation for L-lactate formation was initiated by ceasing air sparging and reducing agitation to 200 rpm when the cell density (OD_600_) reached about 20. During the anaerobic phase, the pH was controlled at 6.5 by the addition of 25% (*w*/*v*) Ca(OH)_2_. The residual glucose concentration was maintained above 3 g/L by adding GOS syrup (980 mL of GOS syrup was added in total). The fermentations were stopped when the glucose was exhausted. The temperature during the aerobic culture stage of cells was 37 °C, and the temperature during the anaerobic fermentation stage was 42 °C.

### 2.5. Analytical Methods

Quantitative analysis of GOS, lactic acid, and related metabolites followed the methods previously described [9,26,28]. Glucose concentration was measured using a glucose biosensor (SBA-40C; Biology Institute of Shandong Academy of Sciences, Jinan, China). Samples were pretreated with H_2_SO_4_ (5% of sample volume) to release organic acids precipitated with CaCO_3_ or Ca(OH)_2_ during fermentation when necessary. Organic acids and ethanol were analyzed by a HPLC (Agilent 1260, Santa Clara, CA, USA) with UV (210 nm) and refractive index detectors using an Aminex HPX-87H column (Bio-Rad, Hercules, CA, USA, 300 × 7.8 mm) with 5 mmol/L H_2_SO_4_ as the eluent (0.6 mL/min; 65 °C; 20 μL injection). The standard curve ranged from 0.2 to 10.0 mg/mL using L-lactic acid, acetic acid, formic acid, and succinic acid standards with ≥98% purity (Sigma-Aldrich, Shanghai, China). Components were identified by comparing retention times with the standards, and contents were calculated by external standard methods. Lactic acid isomeric purity was determined using a chiral column (CLC-L; Advanced Separation Technologies, Whippany, NJ, USA) with 5 mM CuSO_4_ mobile phase (1 mL/min, 25 °C, 20 μL injection) and UV detection at 254 nm [29]. GOS quantification used a HPLC (Agilent 1260, Santa Clara, CA, USA) with RI detector and Sugar-Pak™ I column (Waters, Milford, MA, USA, 6.5 × 300 mm) with water as the mobile phase (0.5 mL/min, 80 °C, 20 μL injection). The standard curve ranged from 0.2 to 10.0 mg/mL using GOS standards (≥98% purity, Sigma-Aldrich). Each component was identified by comparing retention times with the standards, and content was calculated by external standard methods, expressed as percentage of dry weight.

### 2.6. Metabolic Network Analysis of B. licheniformis

Using the *Bacillus licheniformis* DSM13 genome as a reference, analysis was conducted according to the Biocyc Pathway (https://biocyc.org/) (accessed on 20 December 2020).

### 2.7. Data Statistics and Analysis

All experiments were repeated at least three times, and the experimental data were expressed as Mean ± standard deviation (Mean ± SD). Statistical analysis was performed using the SPSS 22.0 software. One-way ANOVA was used to evaluate the significance of differences among groups based on independent biological replicates. A *p* value < 0.05 indicated a statistically significant difference, and Tukey’s honestly significant difference test was employed as the post hoc test [30]. The chart drawing was completed using the Origin 2021 software.

## 3. Results

### 3.1. Metabolic Network Analysis of Pyruvate in B. licheniformis

**Figure 1:**
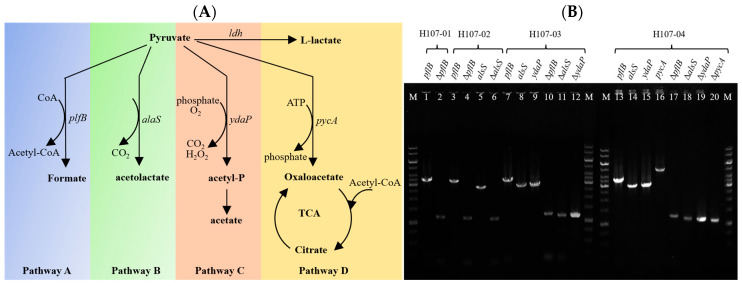
Pyruvate metabolic network and regulation in *B. licheniformis.* (**A**): Analysis of pyruvate metabolic pathways in *B. licheniformis*. (**B**): Deletions of *pflB***,** *alsS***,** *ydaP*, and *pycA* in *B. licheniformis* H107 and diagnostic PCR verification of mutant strains. M: 1 kb DNA Marker; Lanes 1–2: mutations of *pflB* in mutant H107-01; Lanes 3–6: mutations of *pflB* and *alsS* simultaneously occurred in mutant H107-02; Lanes 7–12: mutations of *pflB*, *alsS*, and *ydaP* occurred simultaneously in mutant H107-03; and Lanes 13–20: mutations of *pflB*, *alsS*, *ydaP*, and *pycA* occurred simultaneously.

Based on bioinformatics data, the central carbon metabolic network of strain H107 was reconstructed (Figure 1A). Pyruvate metabolism encompasses multiple branched pathways, including the PflB pathway (pyruvate → formic acid + acetyl-CoA), the AlsS pathway (pyruvate → acetolactate), the YdaP pathway (pyruvate → acetyl phosphate), and the PycA pathway (pyruvate → oxaloacetate). Guided by this analysis, sequential deletions of *pflB*, *alsS*, *ydaP*, and *pycA* genes were performed using homologous recombination techniques [21,22], verified by diagnostic PCR (Figure 1B). The resulting engineered strains were named H107-01 (Δ*pflB*), H107-02 (Δ*pflB*, Δ*alsS*), H107-03 (Δ*pflB*, Δ*alsS*, Δ*ydaP*), and H107-04 (Δ*pflB*, Δ*alsS*, Δ*ydaP*, Δ*pycA*).

The shake-flask fermentation results (Table 2) demonstrated the significant impact of these genetic modifications on metabolic distribution and function. Specifically, the deletion of *pflB* resulted in the complete inhibition of formic acid synthesis and a 79.3% increase in L-lactic acid production. Dual deletion of *pflB* and *alsS* further elevated lactic acid production by 13.5%, although acetate synthesis surged by 127.5% due to residual acetolactate pathways. Furthermore, *ydaP* deletion marginally affected acetic acid levels and improved L-lactic acid production, whereas *pycA* deletion sharply redirected carbon flow, increasing L-lactic acid output by 82.8% relative to H107-03. However, cumulative lactic acid production across these mutants remained suboptimal (0.29–1.06 g/L), necessitating further optimization to achieve industrially viable yields.

### 3.2. Selection of Lactose and GOS Non-Metabolizing Mutants

**Figure 2:**
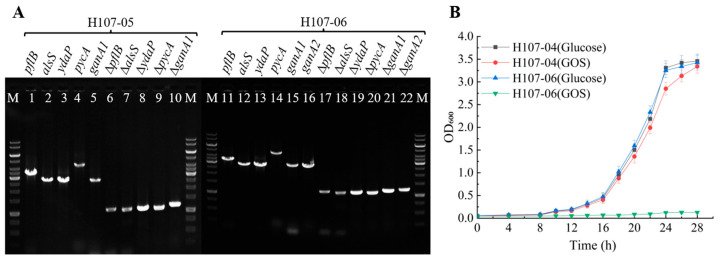
Creation of mutant strain H107-06 and its carbon source utilization characteristics. (**A**): Deletion of *ganA1* and *ganA2* in mutant strain H107-04 and diagnostic PCR verification of the mutant strains; M: 1 kb DNA Marker; lanes 1–10: mutations of *pflB*, *alsS*, *ydaP*, *pycA*, and *ganA1* occurring simultaneously in mutant strain H107-05; lanes 11–22: mutations of *pflB*, *alsS*, *ydaP*, *pycA*, *ganA1*, and *ganA2* occurring simultaneously in mutant strain H107-06. (**B**): Growth characteristics of mutant strains under shake-flask culture.

Bioinformatic analysis identified *ganA1* and *ganA2* as pivotal genes encoding β-galactosidases responsible for GOS and lactose metabolism in strain H107. These genes were sequentially deleted using homologous recombination techniques [21,22] from H107-04 to create H107-05 (Δ*ganA1*) and H107-06 (Δ*ganA1*, Δ*ganA2*). Diagnostic PCR (Figure 2A) confirmed successful deletions, reducing the target gene sizes from 2.9 and 2.8 kb to approximately 1.2 kb each.

Carbon source utilization tests using glucose or GOS (20 g/L) as the sole carbon sources revealed that the mutant H107-06 retained its ability to grow efficiently in glucose (OD_600_: 3.25 ± 0.16 after 24 h) but completely lost its capacity to metabolize GOS (OD_600_: 0.12 ± 0.02). In contrast, its parental strain, H107-04, continued to grow normally in a GOS-based medium (OD_600_: 2.85 ± 0.14). This demonstrates that the dual deletion of *ganA1* and *ganA2* effectively blocks GOS degradation, enabling its selective retention for prebiotic functionality.

### 3.3. Enhancement of L-Lactic Acid Synthesis in B. licheniformis

To enhance the L-lactic acid synthesis capacity, the *BcoaLDH* gene encoding L-lactate dehydrogenase from *B. coagulans* was heterologously expressed in H107-06 using the recombinant plasmid pHY-P43-BcoaLDH (Lifeasible, Shirley, NY, USA). This construct features a strong P43 promoter for constitutive expression along with selection markers for *Bacillus* spp. [31].

Shake-flask fermentation revealed significant advancements: H107-06A (carrying pHY-P43-BcoaLDH) achieved an L-lactic acid yield of 4.45 ± 0.38 g/L (Table 3), representing a 222% improvement over H107-06 (1.06 ± 0.07 g/L). Kinetic evaluation showed a fourfold increase in lactic acid production efficiency, with minimal changes in glucose consumption rates, reflecting enhanced carbon conversion into L-lactic acid. The heterologous BcoaLDH expression likely improved the redox balance by providing an additional NADH oxidation pathway, thereby redirecting carbon flux from competing pathways toward L-lactic acid production. Additionally, the optical purity of the L-lactic acid produced reached up to 99.8%. This metabolic efficiency highlights the beneficial integration of exogenous BcoaLDH into *B. licheniformis*.

### 3.4. Establishment and Optimization of a Complementary Synbiotic Production Process

The synergistic production capability of strain H107-06A was validated in a 5 L fermenter using GOS syrup as the carbon source. This process was optimized to achieve distinct operational phases: an aerobic biomass accumulation phase (0–22 h), a microaerobic acid production phase (22–60 h), and a stationary phase (60–72 h), consistent with established fermentation strategies for maximizing product yields [13,32]. During the acid production phase, reduced agitation and controlled pH effectively redirected metabolic flux toward L-lactic acid synthesis, while minimizing by-product formation [10].

At the end of 72 h, H107-06A produced 42.56 ± 1.85 g/L of L-lactic acid with a productivity of 0.59 ± 0.03 g/(L·h) (Table 4). GOS retention reached 141.89 ± 3.56 g/L, accounting for 75.09 ± 1.85% of total sugars, and active biomass concentration was 3.82 ± 0.15 g/L. These results demonstrate a 14.7-fold improvement in lactic acid production compared to the wild-type strain and highlight the efficacy of the engineered system for simultaneous synergistic production of prebiotics, probiotics, and functional metabolites. This aligns with prior findings on the potential of microbial engineering to enable multifunctional fermentation technologies [33].

**Figure 3:**
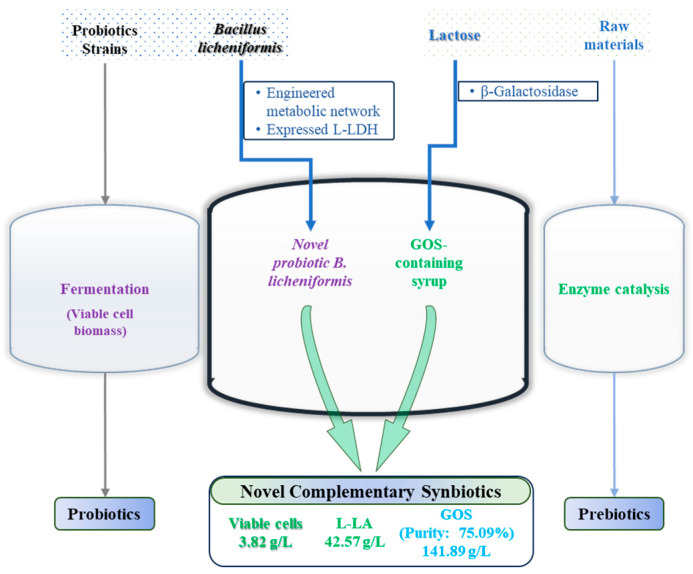
An integrated framework for new complementary synbiotic development. The left and right panels illustrate conventional methods for producing complementary synbiotics, which involves the separate preparation of probiotics and prebiotics prior to mixing. The central panel highlights our innovative approach using metabolically engineered *B. licheniformis* for simultaneous high-yield L-lactic acid production while preserving galactooligosaccharide integrity, enabling efficient complementary synbiotic production in a single integrated process.

Following exploration and research into complementary synbiotic production processes, a next-generation process route for synbiotic development has been proposed (Figure 3). Lactose is enzymatically converted into a GOS syrup using a β-galactosidase with high transglycosylation activity [9]. This syrup serves as the carbon source for *B. licheniformis* fermentation. During this fermentation, the glucose by-product present in the GOS syrup is completely consumed, yielding L-lactic acid and active bacterial biomass, while simultaneously enhancing the purity of the GOS. This technology differs fundamentally from the conventional simple blending of probiotics with GOS. It leverages metabolic engineering technology to achieve the synergistic production of active bacterial cells, high-purity GOS, and functional L-lactic acid, thereby providing an innovative technological pathway for the development of complementary synbiotics.

## 4. Discussion

This study significantly enhanced L-lactic acid synthesis capabilities by systematically deleting pyruvate metabolic branch genes. Comprehensive genetic modifications were performed, including the deletion of *pflB*, *alsS*, *ydaP*, and *pycA*, to redirect carbon flux towards L-lactate production [28,34]. The deletion of *pflB* resulted in a near-complete suppression of formic acid synthesis, while increasing L-lactic acid output by 79% (from 0.29 g/L to 0.52 g/L). Sequential deletions of *alsS* and *ydaP* elevated L-lactate production to 0.58 g/L, albeit with increased acetate accumulation—a potential outcome of intracellular NADH/NAD^+^ balance regulation [32,35]. The final deletion of *pycA* redirected pyruvate flux away from the tricarboxylic acid cycle, yielding L-lactic acid levels of 1.06 g/L—a remarkable step towards industrially relevant production. These findings align with prior insights into the utility of pathway engineering for optimizing microbial metabolite synthesis [36,37].

Moreover, the successful double knockout of genes *ganA1* and *ganA2* effectively blocked GOS degradation pathways while preserving its prebiotic functionality. This innovative approach achieved the selective utilization of glucose in GOS syrup, enhancing substrate purity and fermentation efficiency [9]. The preservation of GOS is critical for synbiotic applications, as it directly contributes to the functional properties of the final product [1].

Anaerobic conditions significantly enhance L-lactic acid production, which is related to the accumulation of NADH and the increased activity of lactate dehydrogenase [38]. During the scale-up process in the fermenter, the L-lactic acid synthesis was further optimized by regulating dissolved oxygen levels and pH. Optimizing the feeding strategy effectively reduced substrate inhibition, raising the yield to 42.56 g/L. Research indicates that maintaining an appropriate glucose concentration (5–10 g/L) is beneficial in reducing by-product accumulation [39,40].

## 5. Study Limitations and Future Perspectives

Although the engineered *B. licheniformis* strain demonstrated promising results at the 5 L scale, several aspects merit further investigation. The metabolic stability of the recombinant strain during prolonged fermentation, possible plasmid loss under industrial conditions, and downstream purification challenges for high-purity GOS require assessment. Future studies could focus on chromosomal integration of BcoaLDH to enhance genetic stability and on multi-omics analyses (transcriptomics and metabolomics) to elucidate global regulatory mechanisms driving L-lactic acid accumulation and GOS retention. Co-culture strategies with the probiotic *Lactobacillus* or *Bifidobacterium* species may also enhance the synbiotic functionality of the final product.

## 6. Conclusions

This study successfully demonstrates the potential of metabolic engineering in *B. licheniformis* for developing innovative complementary synbiotics. By systematically engineering pyruvate metabolic pathways and blocking GOS degradation, the engineered strain H107-06A achieved remarkable improvements in L-lactic acid production (42.56 g/L in a 5 L fermenter), glucose conversion efficiency, and GOS retention (75.09%). These findings establish a scalable and synergistic fermentation platform for producing probiotics, prebiotics, and functional metabolites.

The study provides a scientifically robust framework for overcoming existing limitations in synbiotic production, contributing to advancements in the food and health industries and supporting broader applications in functional nutrition [1,5]. Future work may explore additional pathway optimizations or co-culture systems to further enhance metabolic efficiencies.

## Figures and Tables

**Table 1:** Strains and plasmids used in this study.

Strains/Vectors	Characteristics	Source
Strains		
*E. coli* JM109	*end*A1, *rec*A1, *gyr*A96, *thi*, *hsd*R17, *rel*A1, *sup*E44, λ-, Δ(*lac-pro*AB), [F’, *tra*D36, *pro*AB, laqIqZΔM15]	Lab stock
*B. licheniformis* H107	Wild-type *B. licheniformis*	Lab stock
*B. licheniformis* H107-01	∆*pflB*	This study
*B. licheniformis* H107-02	∆*pflB*, ∆*alsS*	This study
*B. licheniformis* H107-03	∆*pflB*, ∆*alsS*, ∆*ydaP*	This study
*B. licheniformis* H107-04	∆*pflB*, ∆*alsS*, ∆*ydaP*, ∆*pycA*,	This study
*B. licheniformis* H107-04A	∆*pflB*, ∆*alsS*, ∆*ydaP*, ∆*pycA*, pHY-P43*-*BcoaLDH	This study
*B. licheniformis* H107-05	∆*pflB*, ∆*alsS*, ∆*ydaP*, ∆*pycA*, ∆*ganA2*	This study
*B. licheniformis* H107-06	∆*pflB*, ∆*alsS*, ∆*ydaP*, ∆*pyc*A, ∆*ganA2*, ∆*ganA1*	This study
*B. licheniformis* H107-06A	∆*pflB*, ∆*alsS*, ∆*ydaP*, ∆*pycA*, ∆*ganA2*, ∆*ganA1*, pHY-P43-BcoaLDH	This study
Vectors		
pUB-EX	Km^R^, Derived from T2(2)-ori, *E. coli-Bacillus* shµttle vector, thermosensitive replication	[21,22]
pUB-EX-*pflB*	Km^R^, thermosensitive plasmid, used for deletion of *pflB*	This study
pUB-EX-*alsS*	Km^R^, thermosensitive plasmid, used for deletion of *alsS*	This study
pUB-EX-*ydaP*	Km^R^, thermosensitive plasmid, used for deletion of *ydaP*	This study
pUB-EX-*pycA*	Km^R^, thermosensitive plasmid, used for deletion of *pycA*	This study
pUB-EX-*ganA2*	Km^R^, thermosensitive plasmid, used for deletion of *ganA2*	Lab stock
pUB-EX-*ganA1*	Km^R^, thermosensitive plasmid, used for deletion of *ganA1*	Lab stock
pHY-P43-BcoaLDH	Tet^R^, Amp^R^, P43, *BcoaLDH*	[23]

**Table 2:** Distribution of major metabolites in each mutant strain (shake-flask level).

Strains	L-Lactic Acid (g·L^−1^)	Acetic Acid (g·L^−1^)	Formic Acid (g·L^−1^)	Succinic Acid (g·L^−1^)	Biomass (g·L^−1^)
WT	0.29 ± 0.03	0.54 ± 0.03	0.63 ± 0.12	3.75 ± 0.28	1.52 ± 0.11
H107-01	0.52 ± 0.04 *	0.40 ± 0.02 *	ND	2.85 ± 0.21 *	1.43 ± 0.09
H107-02	0.59 ± 0.04 *	0.91 ± 0.06 **	ND	2.66 ± 0.19 *	1.38 ± 0.10
H107-03	0.58 ± 0.03 **	0.85 ± 0.06 **	ND	2.84 ± 0.21 *	1.35 ± 0.08
H107-04	1.06 ± 0.07 **	0.70 ± 0.05 **	ND	3.01 ± 0.22 *	1.31 ± 0.09

ND indicates not detectable. * *p* < 0.05 and ** *p* < 0.01, compared with WT. Fermentation conditions: 20 g/L glucose, 37 °C, and 48 h.

**Table 3:** L-lactic acid fermentation performance of H107-06A under different conditions.

Cultivation Conditions	L-Lactic Acid (g·L^−1^)	Glucose Consumption (g·L^−1^)	Conversion Rate (%)	Biomass (g·L^−1^)
Aerobic	2.52 ± 0.23	8.64 ± 0.46	29.15 ± 1.23	2.13 ± 0.15
Microaerobic	3.24 ± 0.31	9.43 ± 0.49	34.37 ± 1.67	1.82 ± 0.12
Anaerobic	4.45 ± 0.38	9.90 ± 0.52	44.93 ± 1.94	1.57 ± 0.11

The data was the mean ± standard deviation (n = 3). Conversion rate = L-lactic acid production/Glucose consumption ×100%. Fermentation conditions: 20 g/L glucose, 37 °C and 48 h.

**Table 4:** Preparation and comparison of novel complementary synbiotic by strain H107-06A in a 5 L fermenter.

Indicator	Wild Type	H107-06A	Fold Increase
L-Lactic acid (g·L^−1^)	2.89 ± 0.45	42.56 ± 1.85 ***	14.7
Glucose conversion rate (%)	25.38 ± 1.86	85.12 ± 2.35 ***	4.4
Biomass (g·L^−1^)	2.55 ± 0.18	3.82 ± 0.15 ***	1.5
Relative purity of GOS (%)	53.67 ± 2.12	75.09 ± 1.85 ***	1.4

*** *p* < 0.001, compared with WT.

## Data Availability

Data are contained within the article and Supplementary Materials.

## Supplementary Materials

The following supporting information can be downloaded at: https://www.mdpi.com/article/10.3390/microorganisms13112530/s1, Table S1: The oligonucleotide sequence of primers used in this study.
