# Supplementary material for: Metabolic Engineering of Bacillus licheniformis for High-Yield L-Lactic Acid and Galactooligosaccharide Retention in Complementary Synbiotics Production
Source: Microorganisms. 2025 Nov 4;13(11):2530. doi: 10.3390/microorganisms13112530 (PMC12654299; doi:10.3390/microorganisms13112530)
Supplement: Supplementary file 1 [file microorganisms-13-02530-s001.zip › microorganisms-3890516-supplementary.pdf]

Table S1. The oligonucleotide sequence of primers used in this study.

Primer	Nucleotide sequence (5'→3')
pflB-F	CGGAAAGACATTGATTTTCTCC
pflB-R	AATCGGCATCGATATCGGAG
alsS-F	CACGGACGGGAACCGGTTTC
alsS-R	CATTGCGGTCTTCATCTATAAAGTGA
ydaP-F	CGGGTGTGACAAGCGTCGTC
ydaP-R	AATGTGGTGAGATAAATTGCGAG
pycA-F	CCGATTTTATTATGGCGATCATC
pycA-R	ATGGCTTTGCACTTCGGAAT
ganA2-F	CAGATGCTTGGAATGATGAACAG
ganA2-R	GACATAATTGACGCCTGCTTC
ganA1-F	CGTCAATGAAAAAGGGCTGTC
ganA1-R	ACGGCGAAAATACGGAAAAT
